# Real-Time Plant Leaf Counting Using Deep Object Detection Networks

**DOI:** 10.3390/s20236896

**Published:** 2020-12-03

**Authors:** Michael Buzzy, Vaishnavi Thesma, Mohammadreza Davoodi, Javad Mohammadpour Velni

**Affiliations:** School of Electrical & Computer Engineering, University of Georgia, Athens, GA 30602, USA; michael.buzzy25@uga.edu (M.B.); vaishnavi.thesma25@uga.edu (V.T.); mohammadreza.davoodi@uga.edu (M.D.)

**Keywords:** deep learning, plant leaf counting, You Only Look Once (YOLO) network, real-time decision-making

## Abstract

The use of deep neural networks (DNNs) in plant phenotyping has recently received considerable attention. By using DNNs, valuable insights into plant traits can be readily achieved. While these networks have made considerable advances in plant phenotyping, the results are processed too slowly to allow for real-time decision-making. Therefore, being able to perform plant phenotyping computations in real-time has become a critical part of precision agriculture and agricultural informatics. In this work, we utilize state-of-the-art object detection networks to accurately detect, count, and localize plant leaves in real-time. Our work includes the creation of an annotated dataset of *Arabidopsis* plants captured using Cannon Rebel XS camera. These images and annotations have been complied and made publicly available. This dataset is then fed into a Tiny-YOLOv3 network for training. The Tiny-YOLOv3 network is then able to converge and accurately perform real-time localization and counting of the leaves. We also create a simple robotics platform based on an Android phone and iRobot create2 to demonstrate the real-time capabilities of the network in the greenhouse. Additionally, a performance comparison is conducted between Tiny-YOLOv3 and Faster R-CNN. Unlike Tiny-YOLOv3, which is a single network that does localization and identification in a single pass, the Faster R-CNN network requires two steps to do localization and identification. While with Tiny-YOLOv3, inference time, F1 Score, and false positive rate (FPR) are improved compared to Faster R-CNN, other measures such as difference in count (DiC) and AP are worsened. Specifically, for our implementation of Tiny-YOLOv3, the inference time is under 0.01 s, the F1 Score is over 0.94, and the FPR is around 24%. Last, transfer learning using Tiny-YOLOv3 to detect larger leaves on a model trained only on smaller leaves is implemented. The main contributions of the paper are in creating dataset (shared with the research community), as well as the trained Tiny-YOLOv3 network for leaf localization and counting.

## 1. Introduction

Non-destructive, image-based plant phenotyping methods have proven their benefits for quantitative analysis of plant images. Through a combination of new imaging technologies, robotic platforms (ground and aerial vehicles) [1], and novel control algorithms to deploy robotic systems for covering large greenhouses and agricultural fields [2], the capacity to take images of plants and crops has expanded dramatically in the last few years [3]. However, a key requirement for image-based phenotyping tools is to automatically transform those pictures into reliable and accurate phenotypic measurements. In addition, these tools must be capable of measuring a wide variety of phenotypic traits to allow for flexibility and relevance to a wide range of scientific applications.

The number of leaves of a plant is considered to be one of the key phenotypic metrics related to its development and growth stages [4], flowering time [5], and yield potential. The conventional manual counting approach is costly, time-consuming, and laborious. Therefore, various machine learning approaches have been introduced recently to address the leaf counting problem. However, for many reasons, including plants’ rapid growth and leaf occlusion and illumination problems, automatic counting of plant leaves is also a challenging task [6]. In the current machine learning community, deep learning neural networks have become widely used in many image-based tasks such as image classification and object detection and segmentation, among others [7]. Consequently, deep learning methods have recently been utilized to also address this problem, i.e., prediction of the number of leaves.

### 1.1. Related Work

From a machine learning perspective, counting the number of leaves can be classified as belonging to one of the following categories [8]; (i) learning a direct image-to-count regressor model [9], or (ii) obtaining a per-leaf detection and segmentation, which automatically leads to the number of leaves in a rosette [10].

**Counting via direct regression methods:** In these methods, the deep convolutional neural networks are used to integrate image feature extraction with regression in a single pipeline with the goal of leaf counting. In [11], an open source deep learning tool, Deep Plant Phenomics, was introduced which implements deep convolutional neural networks for the purpose of leaf counting, mutant classification, and age regression from top-down images of plant rosettes. The authors of [12] proposed a multi-input deep network, called Pheno-Deep Counter, that combines information coming from different imaging sources and can predict leaf count in rosette-shaped plants.

**Counting via object detection and segmentation methods:** There exists a number of works that implement object detection or segmentation networks in order to address the leaf counting problem. Object detection algorithms operate by simultaneously preforming object classification, as well as localization. They can do this in a single pass or use multiple networks in conjunction with one another. These networks offer superior accuracy as they count leaves on a leaf by leaf basis rather than from a whole plant perspective. There are several approaches within the field of object detection and localization, each with different strengths and weaknesses. Networks like You Only Look Once (YOLO) [13] detect objects quickly but struggle with densely packed groups of objects. Conversely, networks like Region-based Convolutional Neural Network (R-CNN) [14] are slower but can more easily discern tight groups of objects; however, the amorphous shapes of leaves also can lead to double detection of a leaf. Object detection and segmentation approaches give the unique opportunity to experiment with the pros and cons of different network structures to a greater degree than with the direct regression methods.

In [15], a Recurrent Neural Network (RNN) architecture with an attention mechanism was proposed to compute instance segmentation jointly with counting. The performance of the method was shown on the CVPPP plant leaf dataset [16], as one of the instance segmentation benchmarks. In [17], the Mask R-CNN method was used for leaf segmentation and counting. The authors of [18] proposed a data augmentation method, preserving the photorealistic appearance of plant leaves. The augmented data was then used as training set for a Mask R-CNN network.

### 1.2. Contributions of This Work

In most of the existing platforms and current literature, collected images of the plants are stored locally and later transferred to hard disks and processed offline for the purpose of decision-making (e.g., counting leaves). However, it is critical to develop platforms that can automatically collect and analyze images in real-time for leaf counting. To achieve this goal, we design a robotic platform which is capable of navigating between plant rows, capturing the top-view images, and then detecting and counting the number of leaves in real-time. We adapt, train, and apply a Tiny-YOLOv3 model to accurately count leaves in images acquired with our robotic platform. Using our trained model, a complete list of locations and dimensions of bounding boxes are generated to identify and count the number of leaves in images. Along with the YOLO model, we also provide a comparison with another state-of-the-art object detection method, namely, Faster R-CNN.

The second contribution of this work is the release of our training and testing data sets. The images used in the dataset have been captured over the course of one month from a group of 60 *Arabidopsis* plants and taken using a high-quality DSLR camera from a top down perspective. To obtain the labeled data, the images were taken and then each leaf was labeled with a bounding box representing its location.

The third contribution of this work lies in the implementation of transfer learning using Tiny-YOLOv3 to detect larger, mature leaves of the *Arabidopsis* plant, without retraining the entire model from scratch. The model is first trained on images with smaller leaves, organized by timestamp. This trained model is then used to detect, localize, and count larger leaves.

The remainder of this paper is structured as follows. In Section 2, the structure of our proposed leaf counting network, Tiny-YOLOv3, as well as our proposed experimental setup are described. In Section 3, the generated dataset, image processing and labeling, training procedure, as well as the implementation details are provided. Finally, in Section 4, concluding remarks and directions for future research works are discussed.

## 2. Our Approach to Leaf Detection and Counting

### 2.1. Deep Object Detection Model

**Figure 1:**
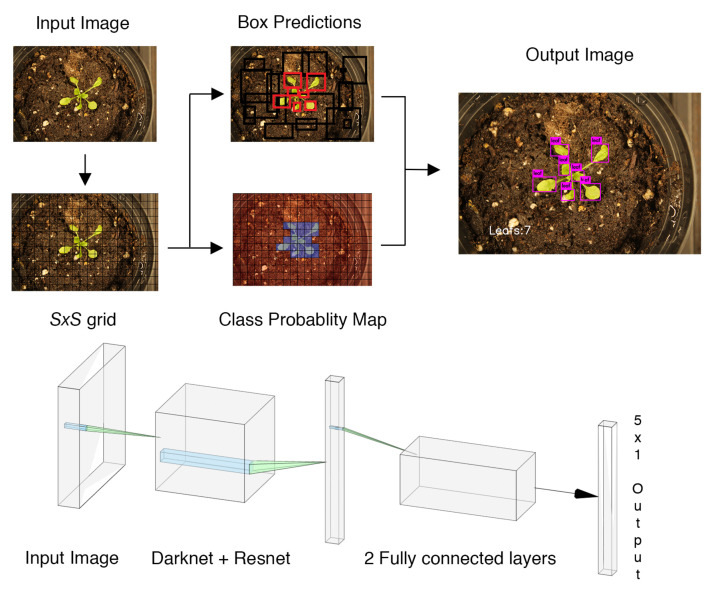
You Only Look Once (YOLO) framework taking an image as input into a deep convolutional neural network and outputs the leaf detection, where the bottom part of the figure consists of a diagram of the YOLO network architecture.

You Only Look Once (YOLO) is a state-of-the-art object detection and localization network [13]. It is a single network that does localization and identification in a single pass. YOLO splits up an input image into an S×S grid. For each square in the grid, the network proposes a possible bounding box and a confidence score for how certain the network is that the box contains an object. The network also creates a class probability map for each cell in this S×S grid. The output of the network is then any proposed bounding box with a confidence score above a threshold value and the class that coincides with that bounding box on the class probability map. The YOLO-based leaf counting network is shown in Figure 1.

YOLO has been very recently used by other researchers for plant phenotypic detection, localization, and segmentation. For example, YOLO has been used to detect fruits such as apples and pears and had still been successful for fruit detection even when fruit was smaller than its foilage or even similar in color to its foilage [19]. Additionally in [20], YOLO has been used for rice plant tip detection to ensure high yield for rice-dependent countries in Asia. Furthermore, YOLO has even been used for weed localization and segmentation to ensure better yield for sugar beets [21]. In addition, some recent works have modified the architecture of YOLO to better perform on their custom datasets. For example, the authors of [22] used features from YOLOv3 and Tiny-YOLOv2 to create a custom architecture to detect mango fruits in trees and coined their new architecture as “MangoYOLO”. Their results in using the MangoYOLO architecture yielded in high accuracy in terms of F1 Score, AP, and inference time.

YOLOv3 [23] is the third generation of the YOLO architecture. For leaf counting, the choice of this version is very significant. Compared to its predecessors, YOLOv3 creates three grids instead of one. Each of these grids is composed of a different size mesh. Previous YOLO generations struggle at object detection of close, densely packed objects [13]. Leaves quite often are found clumped together or overlapping. The use of the finer mesh allows the network to better detect those densely clumped leaves.

In this work, we opted and used the Tiny-YOLOv3 [24]. This version of YOLO uses fewer layers than the full YOLO network and is able to run faster at the cost of accuracy. For our leaf counting algorithm, the possible loss in accuracy is less pronounced due to the fact that there is only one category (i.e., leaf) and that all the objects in that category are primarily primitive shapes with similar structures. Another benefit to using Tiny-YOLOv3 is its ability to run on lower end hardware. This makes the implementation of the network more accessible and more versatile. We later demonstrate this versatility by getting near-real-time performance of a budget Android phone.

We chose to train and evaluate our network using the Darknet [25] deep learning framework because of its speed and portability. Being written in C, Darknet could be deployed on any device with a C compiler quickly and easily. Beyond Darknet, we also added a Python script to watch the output of Darknet and enumerate the number of generated bounding boxes. Moreover, OpenCV library was used to add text (print the final number of leaves) to the final images.

### 2.2. Experimental Setup

**Figure 2:**
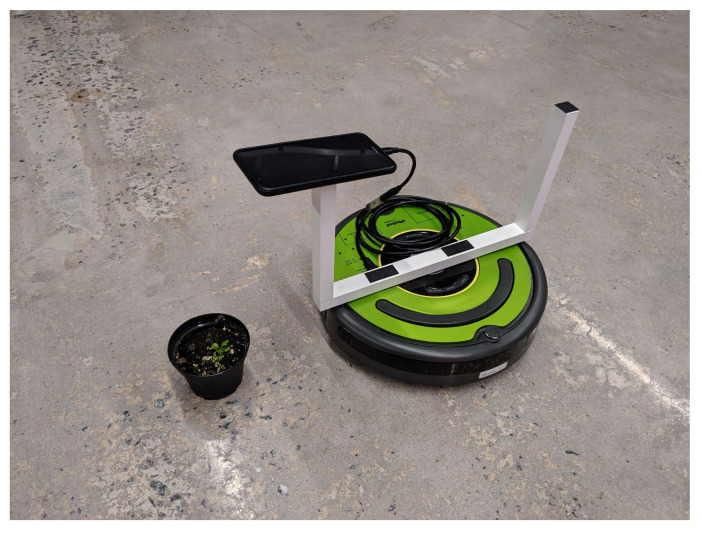
Deployed platform for data acquisition and real-time data processing.

In our platform, an Honor 7× Android phone was attached to an iRobot Create2 [26], as shown in Figure 2. The Android phone was responsible for capturing the images with its on-board camera and preforming the computations required for the leaf counting network. We note that the Android phone was not used for creating the training and testing datasets, but rather was used to deploy the completed experiment in a controlled environment. This experimental setup shows the versatility of using Darknet and Tiny-YOLOv3, as our network is able to be deployed on very low cost hardware effectively. On the Android phone, the inference time for the network averaged around 5 s. The robot is able to follow a predefined path and preform a near-real-time analysis on several plants arranged in a typical row pattern that would be found in a greenhouse setting. With better hardware, the network could easily preform more challenging missions, but this demo shows a proof of concept.

## 3. Experiments and Results

**Figure 3:**
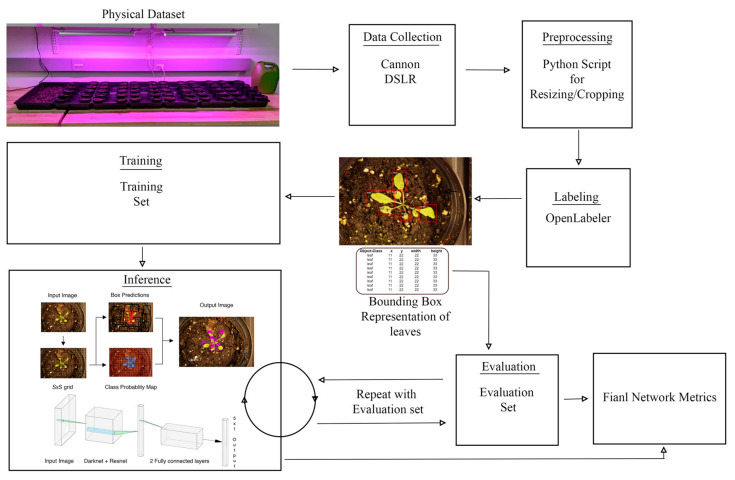
The general pipeline of our proposed architecture from generating the dataset to training the model. Different blocks are explained with more details in the ensuing section.

The whole pipeline of our proposed architecture is illustrated in Figure 3 and will be described with more details in the following subsections.

### 3.1. Dataset

The candidate plant for our data set was *Arabidopsis thaliana* [27]. *Arabidopsis* was chosen for the following reasons. It is easy, very fast, and inexpensive to grow, and produces many seeds. Gathering large amounts of data in a reasonable time frame was a key factor in plant choice; by choosing a quickly growing plant, we were able to construct the dataset in only a few weeks. Finally, we chose *Arabidopsis* for its tolerance to cold temperatures. By choosing a plant robust to cooler temperatures, we were able to build our dataset indoors and did not require any farm or greenhouse (controlled environment) space.

Our *Arabidopsis* plants were grown indoors under red/blue LED grow lamps as shown in Figure 3. The plants were grown in 10×6 plant batches for a total of 60 plants. The plants were watered every other day. The plants were photographed with a Cannon Rebel XS and stored in JPG format. The Canon camera was used to build the training and test datasets to analyze the performance of the YOLO and Faster R-CNN models. The grow lamps were kept on 24/7 to speed up the growth rate.

**Figure 4:**
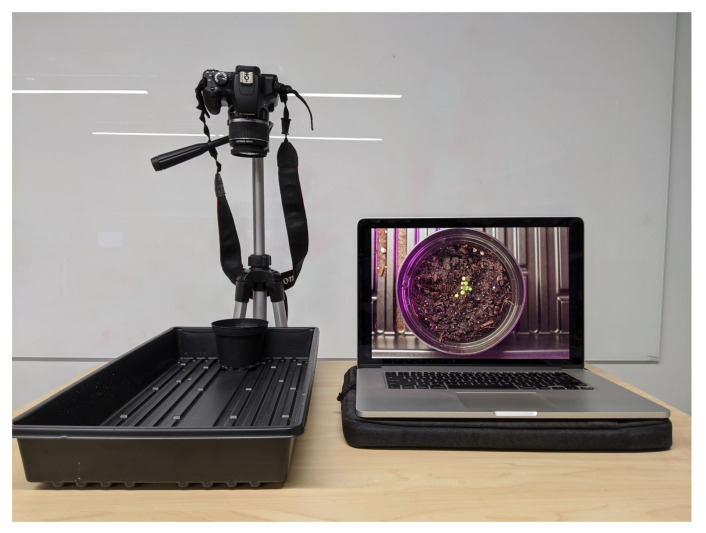
Data ingest station.

The data collection was done daily for weekdays only. The data collection period spanned a period of four weeks. Data collection began when the first leaves became visible and ended once the plants began to shoot up vertical stems for flower growth. Each plant was photographed individually from about six inches away, as shown in Figure 4. The camera settings were as follows; 1/5”, F5.6, ISO800, and the camera was manually focused. Each plant was photographed twice per day ensuring that each photograph featured a distinct rotation and position.

To create the evaluation dataset, we grew another group of plants the same way as in the creation of the training dataset. We found this necessary because we were concerned about the datasets ability to generalize beyond the 60 plants used to create the training dataset. Using a new batch of plants ensured that any bias towards the individual plants in the dataset was avoided.

### 3.2. Image Preprocessing

Images were preprocessed in batches that coincided with the day they were taken. This was to ensure that any settings adjusted were functional for all the plants at that stage of growth. The first step in preprocessing was to crop each image to increase the leaf size relative to the whole picture. Each batch was cropped the same amount, but as the leaves got bigger and naturally filled the frame the cropping factor was decreased. The first batch of images was cropped by a factor of 0.5 in both axes and the last batch was not cropped at all. The next step in preprocessing was to pad each image to make it square. The images were padded with zeros. Finally, each image was downsampled with OpenCV library in Python to a size of 410×410.

### 3.3. Data Labeling

**Figure 5:**
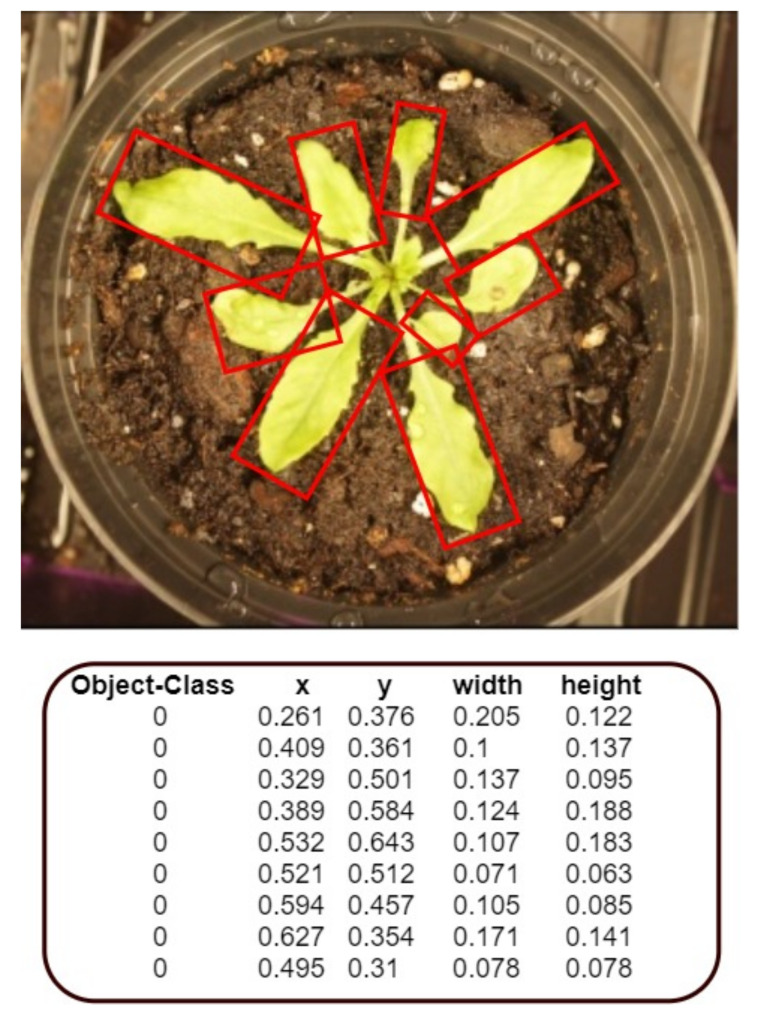
One sample of plant and its annotation from our generated dataset.

The images were labeled using OpenLabeler and the outputs were saved into an XML file using the VOC format. This allowed for easy conversion into other formats to utilize our dataset for various networks. Bounding boxes were drawn around every leaf. If the human labeler was ever uncertain about the bounding of a leaf, the default decision was to draw the box in a way that resulted in fewer leaves rather than more. For each of the labeled images, an additional text file was also generated that contained the annotated bounding boxes’ coordinates, as shown in Figure 5.

The final dataset contains 1000 labeled images of labeled *Arabidopsis* plants containing several thousand labeled leaves. The evaluation set contains 36 labeled *Arabidopsis* plants.

### 3.4. Training Procedure

The best preforming Tiny-YOLO model was trained for a total of 160,000 batches over the course of two days. We preformed the training using a batch size of 24 with subdivisions of 8 in order to accommodate our low GPU memory. Other hyperparameters include momentum of 0.9, weight decay of 0.0005, burn in of 1000, and a learning rate of 0.001.

### 3.5. Implementation Details

**Figure 6:**
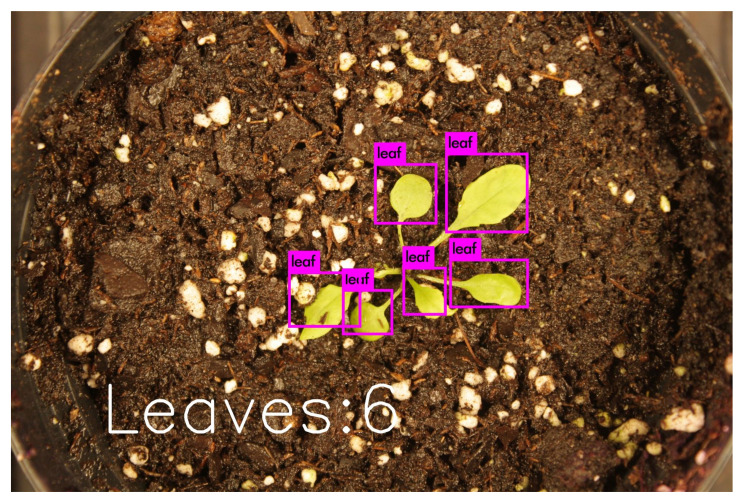
The leaf counting output.

We trained our deep neural network models using Darknet. The training was done on a Quadro P2000 with 5 GB of GDDR5 Memory. The CPU is an Intel CORE i7-7800x with 32 GB of memory. To demonstrate the efficacy of the developed platform and proposed model, we considered a greenhouse lab setup using the plants in test dataset. The robot autonomously navigates between the plants, captures pictures and runs inference to predict the number of leaves in each picture. An illustrative example of such processing and final outcome is shown in Figure 6.

### 3.6. Evaluation Metrics

To evaluate the effectiveness and performance of our proposed approach, the following evaluation metrics, as in [9] (now a consensus in the broad community), are utilized.

(i) Difference in count (DiC) = $\frac{1}{N}\sum_{i=1}^{N}\epsilon_{i};$
(ii) Absolute difference in count (|DiC|) = $\frac{1}{N}\sum_{i=1}^{N}\left|\epsilon_{i}\right|;$
(iii) Mean squared error (MSE) = $\frac{1}{N}\sum_{i=1}^{N}\epsilon_{i}^{2};$
(iv) Percentage agreement (%) = $\frac{1}{N}\sum_{i=1}^{N}1\left[\epsilon_{i}=0\right];$

where $\epsilon_{i}=y_{i}-{\hat{y}}_{i}$ is the difference between the ground truth and algorithmic prediction (number of the estimated instances) and 1[·] is the indicator function, which returns zero if the error $\epsilon_{i}\neq 0$, otherwise returns one [28].

Moreover, the final output of the proposed object detection model is a list of bounding boxes that would ideally contain all of the leaves in an image and their relative locations. The main objective is that the number of boxes accurately match the number of leaves in an image. Denoting boxes as leaf or non-leaf can lead to the following potential scenarios; true positive (*TP*)—correctly classifying a region as a leaf; false positive (*FP*)—incorrectly classifying a background region as a leaf, as well as multiple detection of the same leaf; and false negative (*FN*)—incorrectly classifying a leaf as a background region. In order to quantify *TP*, *FP*, and *FN*, the average precision (AP) metric over the intersection over union (IOU) threshold of 0.5 is utilized. Note that IOU is defined as the intersection area of predicted and ground truth bounding boxes divided by the union area, which quantifies how close the predicted results are to ground truth labels. A threshold above 0.5 on IOU is considered as positive, while under 0.5 is considered as poor detection. It is the area under the recall–precision curve [29], where

(1) $$\begin{array}{cc} \mathrm{precision} & =\frac{TP}{TP+FP}, \end{array}$$

(2) $$\begin{array}{cc} \mathrm{recall} & =\frac{TP}{TP+FN}. \end{array}$$

**Figure 7:**
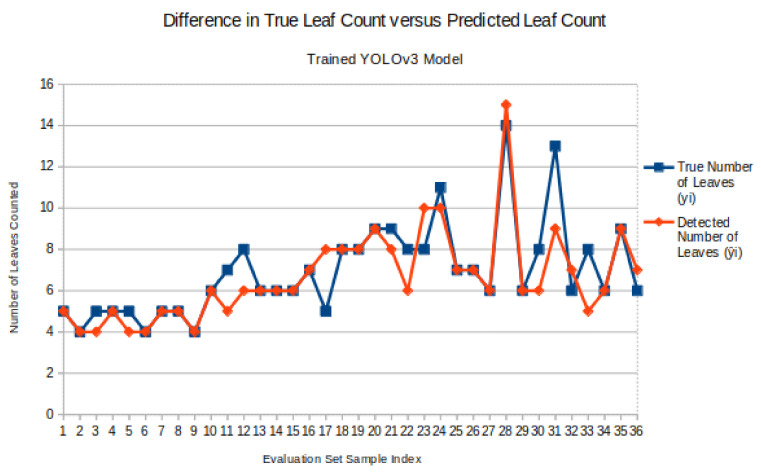
Scatter plot comparison between true leaf count vs. estimated leaf count from YOLO model.

To visually compare the trained YOLO model’s performance on how well it detected leaves, Figure 7 shows a lined scatter plot comparing the true number of leaves (blue) versus the detected number of leaves (red) in an image in the evaluation dataset. To clearly see where the blue and red points overlap, the points are lined and connected. Where both lines overlap at a point indicate that the model was correctly able to identify the true number of leaves. If the red line, which represents the algorithmic prediction, does not align with the blue line, which is the ground truth, then the model may have overestimated or underestimated the true leaf count.

In addition to computing precision and recall, the average accuracy of the models’ performance can be evaluated as

(3) $$\mathrm{accuracy}=\frac{TP+TN}{TP+TN+FP+FN}.$$

Using the precision and recall metrics above, the F1 score can be calculated as another way to analyze the accuracy of a model. Below is the formula for evaluating F1 score in terms of precision and recall.

(4) $$F1\;\mathrm{Score}=\frac{2*precision*recall}{precision+recall}.$$

Additionally, the true positive rate (TPR) and false positive rate (FPR) are also calculated. These metrics allow for a better insight to how well the trained model can correctly identify and localize the leaves in an image, and how often double detection or falsely identifying background as leaves occur, respectively. Ideally, the TPR should be very close to 100% and the FPR should be low and close to 0%.

Finally, the inference time will be utilized to measure the computational complexity. The inference time will be considered as the time it takes for the network to process one image. The time taken to load or initialize the network would not be taken into account.

### 3.7. Final Results and Comparison with Faster R-CNN

The above evaluation metrics obtained using our platform are summarized in Table 1.

We further compare the results of the trained Tiny-YOLOv3 model with another state-of-the-art object detection method, Faster Region-based Convolutional Neural Networks (R-CNN) [14]. Faster R-CNN is near real-time, as the network uses a Region Proposal Network (RPN) to produce object proposals from an input image without using selective search to extract region proposals like R-CNN [30] and Fast R-CNN [31]. This feature greatly reduces the computational time, as selective search algorithm takes a long time to generate region proposals.

The Faster R-CNN network has two main parts: the first being the RPN, which is a full convolutional network, that produces the region proposals on an input image; the second being the Fast R-CNN detector that classifies the region proposals from the RPN. As YOLO is a single network that does localization and identification in a single pass, we expect Faster R-CNN to result in slower training and inference time, and would have comparable performance in accuracy since Faster R-CNN can detect tight group of objects.

It is important to note that to obtain the results shown in Table 1, we used the same Quadro P2000 desktop (that was also employed for training) to run both Tiny-YOLOv3 and Faster R-CNN networks on the test dataset. The Quadro P2000 desktop has 5 GB of GDDR5 Memory and the CPU is an Intel CORE i7-7800x with 32 GB of memory. First, the RPN is trained for 50 epochs for 1000 iterations per epoch. Then, its proposals are used to train the Fast R-CNN network for 50 epochs for 500 iterations per epoch. The total training time was approximately 2.5 days. Training the RPN before the detection network takes a longer amount of time to train since two networks must be trained separately, but an improved accuracy can be achieved.

As it is clear from Table 1, the results of Tiny-YOLOv3 indicate that there is better overall real-time counting of leaves. Specifically, using YOLO, a lower mean-squared error (MSE), higher F1 Score, higher TPR, and lower FPR are achieved compared to Faster R-CNN. While Faster R-CNN provided a higher AP score, which indicates an increased ability to perform object detection and localization, Tiny-YOLOv3 has a significantly lower |DiC| value. This also shows that Tiny-YOLOv3 has higher accuracy and less false positive occurrences than Faster R-CNN. Last, Tiny-YOLOv3 can more quickly detect and localize leaves compared to Faster R-CNN, nearly 100 times faster. As such, Tiny-YOLOv3 can be deployed in the field to analyze plant condition in terms of leaf count in real time.

### 3.8. Transfer Learning of Models with New Datasets

In addition to training the Tiny-YOLOv3 network to detect leaves grown by the *Arabidopsis thaliana* plant, we also implemented a transfer learning method via Tiny-YOLOv3. The source task was to detect smaller leaves that were grown in the plants’ early stages and the target task was to detect larger leaves that were grown in the plants’ later stages, or more mature stages. The goal was to limit the time in retraining the model from scratch as the overall domain, the *Arabidopsis thaliana* plant leaves, changed in size over time as the plant grew.

To accomplish transfer learning, we partitioned our original dataset into two main domains: the source domain and target domain, organized by timestamp. The source domain contained a total of 600 images that were further divided into a training and testing set, with 480 and 120 images, respectively. Similarly, the target domain contained a total of 100 images divided into a training and testing set, with 80 and 20 images, respectively.

The source training set was trained for a total of 160,000 batches, with a batch size of 24, subdivisions of 8, momentum of 0.9, weight decay of 0.0005, burn in of 100, and a low learning rate of 0.001. The evaluation metrics obtained from using this platform are shown in Table 2. The source trained model was tested on the 100 target training images to validate that the two source and target domains are different. Additionally, the source model should result in slightly worse results in the target training set if the two domains are different. The evaluation metrics obtained from using this platform are shown in Table 3.

Based on the results in Table 2 and Table 3, it is observed that there is a slight reduction in accuracy and performance between the trained source model on its own test data and the target training data. Specifically, the absolute difference in count is nearly doubled and the false positive rate (FPR) is also doubled. Moreover, the F1 Score decreased slightly. This signifies that the source and target domains are slightly different but still similar. Thus, we can proceed to retrain and fine-tune the source model to perform better when presented with larger and more mature leaves.

The target training set was trained for an additional 10,000 batches on the already trained source model, for a total of 170,000 batches. By retraining all layers of the source model on the target training set, the model has more flexibility to improve its overall accuracy while not being trained for a long period of time. The total retraining time was less than 2 h. The evaluation metrics obtained from using this platform are shown in Table 4.

From the results given in Table 4, it is observed that there is a significant reduction in the mean-squared error (MSE) and FPR. This indicates that the model that was trained via transfer learning results in better detection of leaves that are actually present in the image. Thus, we can see that transfer learning does indeed result in better detection and localization of smaller, young leaves, and larger, older leaves. As such, training time can be significantly reduced even if the dataset is modified with the addition of new and similar images, as the original trained model can be used.

### 3.9. Discussion

In this work, an autonomous robotic platform was developed which is capable of predicting the number of leaves in real-time. However, the proposed approach has other potential applications in developing an intelligent system to help farm and greenhouse practitioners (experts and non-experts) to capture images of plants with mobile phones and provide *real-time information* about them.

It is noted that the growth conditions of the *Arabidopsis thaliana* for both the test set and the evaluation set were identical. The plants were grown indoor under LED lamps and were all watered on the same frequency. As such, there are small to no differences between each plant in terms of light reception, damage, insect infestation, and mineral nutrition. Thus, the model is biased towards the plants having constant and identical growing conditions. If additional plants of the same species were grown either outdoors with natural light, without LED lamps, or with different mineral nutrition, then the plants may have produced a variety of growth patterns. This variation would result in the model trying to detect potential differences in leaf shape, count, and even damage. As such, the model may fail due to plant condition variation and thereby would be subject to further performance analysis.

Additionally, we only tested one type of plant in this experiment. This species of plant is small, round, and their leaves are easily spread out and quite easy to localize, and leaf shape is larger in comparison to the overall plant size. If a different plant was used with different leaf shape, size, and clustering behavior, then the model would also be subject to different performance or even failure, and would allow for further performance analysis on the model’s ability for learning different plants’ leaves [21].

Consequently, this proof-of-concept study can be eventually used in a wide range of applications by stakeholders ranging from farmers (to estimate the number of leaves and evaluate the plant growth stage and final yield) to agricultural researchers (working to improve crops).

## 4. Conclusions

In this work, we built an autonomous ground robotic device which is capable of near real-time leaf detection and counting. It was demonstrated that given a moderate amount of data on top-view images of plants, our trained model, Tiny-YOLOv3, is able to learn to localize and predict the number of plant’s leaves without any prior knowledge on that specific plant. We made our generated dataset publicly available, with the goal of promoting the use of object detection deep learning models within the plant phenotyping community.

Our ongoing work focuses on creating more robust algorithms to help automate the leaf counting process. By using more powerful platforms with more computational resources, as well as more powerful cameras, we aim to create an autonomous plant phenotyping system. Our solution will be able to first perform localization in real-time to detect potential crops to monitor. The system will then navigate the unknown terrains and map them while providing real-time feedback on the state of the crops in question. This information can then be processed on-board the robot in real-time and either be used to deploy another robot to address any issues or to farm managers for manual/visual inspection.

## Figures and Tables

**Table 1:** Network evaluation metrics.

Metric	Tiny-YOLOv3	Faster R-CNN
DiC	0.25	0.0556
|DiC|	0.8056	1.2778
MSE	2.0833	2.8889
%Agreement	56%	27.78%
AP (@.5)	0.583	0.600
Accuracy	0.88846	0.83088
Precision	0.97059	0.91129
F1 Score	0.94467	0.89866
TPR	91.304%	90.4%
FPR	24.138%	47.826%
Inference time (s)	0.009225	0.917535

**Table 2:** Source domain network evaluation metrics.

Metric	Tiny-YOLOv3
|DiC|	0.575
MSE	1.075
TPR (%)	93.4%
FPR (%)	11.7%
F1 Score	0.961

**Table 3:** Network evaluation metrics for testing source and target differences.

Metric	Tiny-YOLOv3
|DiC|	0.938
MSE	1.788
TPR (%)	91%
FPR (%)	23%
F1 Score	0.94

**Table 4:** Target domain network evaluation metrics.

Metric	Tiny-YOLOv3
|DiC|	1.15
MSE	1.15
TPR (%)	87%
FPR (%)	5%
F1 Score	0.93

## Abbreviations

The following abbreviations are used in this manuscript:

DiC	Difference in count
DNN	Deep neural networks
MSE	Mean-squared error
RNN	Recurrent neural network
R-CNN	Region-based convolutional neural network
YOLO	You only look once
